# Hypoxia on the Expression of Hepatoma Upregulated Protein in Prostate Cancer Cells

**DOI:** 10.3389/fonc.2016.00144

**Published:** 2016-06-15

**Authors:** Ingrid Espinoza, Marcelo J. Sakiyama, Tangeng Ma, Logan Fair, Xinchun Zhou, Mohamed Hassan, Jovanny Zabaleta, Christian R. Gomez

**Affiliations:** ^1^Cancer Institute, University of Mississippi Medical Center, Jackson, MS, USA; ^2^Department of Preventive Medicine, University of Mississippi Medical Center, Jackson, MS, USA; ^3^Department of Biochemistry, University of Mississippi Medical Center, Jackson, MS, USA; ^4^Department of Pathology, University of Mississippi Medical Center, Jackson, MS, USA; ^5^CAPES Foundation, Ministry of Education of Brazil, Brasília, Brazil; ^6^School of Medicine, University of Mississippi Medical Center, Jackson, MS, USA; ^7^Stanley S. Scott Cancer Center, Louisiana State University Health Sciences Center, New Orleans, LA, USA; ^8^Department of Pediatrics, Louisiana State University Health Sciences Center, New Orleans, LA, USA; ^9^Department of Radiation Oncology, University of Mississippi Medical Center, Jackson, MS, USA

**Keywords:** HURP, hypoxia, prostate cancer, LNCaP, C4-2B, tumor

## Abstract

Hepatoma upregulated protein (HURP) is a multifunctional protein with clinical promise. This protein has been demonstrated to be a predictive marker for the outcome in high-risk prostate cancer (PCa) patients, besides being a resistance factor in PCa. Although changes in oxygen tension (pO_2_) are associated with PCa aggressiveness, the role of hypoxia in the regulation of tumor progression genes such as HURP has not yet been described. We hypothesized that pO_2_ alteration is involved in the regulation of HURP expression in PCa cells. In the present study, PCa cells were incubated at 2% O_2_ (hypoxia) and 20% O_2_ (normoxia) conditions. Hypoxia reduced cell growth rate of PCa cells, when compared to the growth rate of cells cultured under normoxia (*p* < 0.05). The decrease in cell viability was accompanied by fivefold (*p* < 0.05) elevated rate of vascular endothelial growth factor (VEGF) release. The expression of VEGF and the hypoxia-inducible metabolic enzyme carbonic anhydrase 9 were elevated maximally nearly 61-fold and 200-fold, respectively (*p* < 0.05). Noted in two cell lines (LNCaP and C4-2B) and independent of the oxygen levels, HURP expression assessed at both mRNA and protein levels was reduced. However, the decrease was more pronounced in cells cultured under hypoxia (*p* < 0.05). Interestingly, the analysis of patients’ specimens by Western blot revealed a marked increase of HURP protein (fivefold), when compared to control (cystoprostatectomy) tissue (*p* < 0.05). Immunohistochemistry analysis showed an increase in the immunostaining intensity of HURP and the hypoxia-sensitive molecules, hypoxia-inducible factor 1-alpha (HIF-1α), VEGF, and heat-shock protein 60 (HSP60) in association with tumor grade. The data also suggested a redistribution of subcellular localization for HURP and HIF-1α from the nucleus to the cytoplasmic compartment in relation to increasing tumor grade. Analysis of HURP Promoter for HIF-1-binding sites revealed presence of four putative HIF binding sites on the promoter of *DLGAP5/HURP* gene in the non-translated region upstream from the start codon, suggesting association between HIF-1α and the regulation of HURP protein. Taken together, our findings suggest a modulatory role of hypoxia on the expression of HURP. Additionally our results provide basis for utilization of tumor-associated molecules as predictors of aggressive PCa.

## Introduction

Prostate cancer (PCa) remains the most common form of cancer affecting men in the Western Hemisphere. In 2015, an estimated 220,800 new cases of PCa are expected to occur in the US and an estimated 27,540 deaths are expected nationwide (1). On the assessment of the aggressiveness of PCa, factors of tumor microenvironment have received increasing attention. In particular, the role of oxygen tension [potentia oxygenii (pO_2_)] in tumor biology is of special relevance. Unappreciated for a long time, tumor hypoxia has been recently linked to malignant progression, metastasis, resistance to therapy, and poor clinical outcomes, particularly in the case of PCa (2–4). Likewise, addition of hypoxia as a variable improved prognostic accuracy of aggressive PCa, when added to currently used clinicopathological variables (5). This demonstrates the relevance of hypoxia, a variable of the microenvironment, as a factor of aggressiveness in PCa.

We previously reported that a subset of transcripts of hypoxia-associated genes are relevant as markers of PCa progression (6). One of these genes, namely hepatoma upregulated protein (HURP), was found to be associated with Gleason score and systemic progression of PCa, in addition to being a potential independent outcome predictor in high-risk PCa (6). Moreover, the induction of HURP expression in PCa cells was shown by us to inhibit γ-irradiation-induced apoptosis *via* destabilization of p53 and ATM, key proteins in the modulation of γ-irradiation-induced apoptosis (7). Thus, in addition to its reliability as a prognostic biomarker in patients at high-risk of developing aggressive PCa, HURP seems to trigger PCa resistance to standard antitumor therapies.

It has been generally accepted that conditions of tumor microenvironment including hypoxia promote disease progression and metastasis *via* mechanisms mediated by chromosomal instability, gene amplification, and decreasing tumor sensitivity to DNA damaging agents (8, 9). The above mentioned tumor-associated aberrations contribute to disease development and resistance to therapies (10–12). HURP expression is tightly regulated during cell cycle progression (13–15) and is a component of the Ran-importin β-regulated spindle assembly pathway (16). HURP possesses significant regions of positive charge that are postulated to interact with microtubules (17), suggesting an essential role for this protein on the regulation of cell cycle control. Accordingly, the overexpression of HURP in 293T cells and NIH3T3 embryonic fibroblasts at low serum levels was associated with the promotion of cell growth and colony formation, respectively (14, 18, 19). In contrast, the knockdown of HURP in SK-Hep-1-derived hepatoma model delayed tumor formation (19).

When analyzed in total, the available information utilizing *in vitro* and *in vivo* models suggest that HURP’s biological properties are compatible with its role in carcinogenesis. Changes in pO_2_ contribute to the aggressiveness of tumors as well, but it is less clear whether hypoxia affects HURP expression. The present study provides the first insight into the biological properties of HURP as a hypoxia-associated gene in tumor development and progression.

## Materials and Methods

### Cell Culture

The human PCa cell lines LNCaP and C4-2B were obtained from the Characterized Cell Line Core Facility, University of Texas MD Anderson Cancer Center. C4-2B cells represent a human bone metastatic PCa and were derived from LNCaP cells. They have more aggressive characteristics when compared to their parental cells (20). For methylation experiments, we utilized LNCaP, DU-145, and PC3 PCa cell lines purchased from the ATCC. Cells were cultured as recommended by the company. All utilized cell lines were genotyped by STR DNA fingerprinting. They were mycoplasma-free following the detection with the MycoAlert™ Mycoplasma Detection Kit (cat # LT07-218; Lonza, Allendale, NJ, USA). Cells were routinely cultured in phenol red RPMI-1640 supplemented with 10% heat-inactivated fetal bovine serum (Cellgro, Manassas, VA, USA) at 37°C in humidified air enriched with 5% CO_2_ and with O_2_ content either 20% (normoxic) or 2% (hypoxic) in a CB-150 (Binder, Germany) CO_2_ incubator. The cells were trypsinized at 80–90% confluence and plated at the density of 12,000 cells/cm^2^. The medium was not refreshed during the course of the experiments. To evaluate cell viability, cells excluding trypan blue were counted by the aid of a hemocytometer.

### Measurement of VEGF Concentration in Conditioned Media

Concentrations of vascular endothelial growth factor (VEGF) in supernatants were measured by a commercial enzyme-linked immunosorbent assay (ELISA) kit (R&D Systems, Inc., Minneapolis, MN, USA) according to the manufacturer’s instructions. This ELISA kit has been shown to recognize recombinant human VEGF_165_, recombinant human VEGF_121_, and recombinant human VEGF_165b_. The lower detection limit of the kit was 31.3 pg/mL. Rate of VEGF secretion was expressed as pg/(mL/cell/day).

### Quantitative RT-PCR

Total mRNA was isolated using RNeasy Mini kit (Qiagen, Germantown, MD, USA) according to the manufacturer’s instructions: 1-μg RNA was reversely transcribed using SuperScript III First Strand Synthesis (Invitrogen, Grand Island, NY, USA). Subsequently, quantitative PCR was performed with a LightCycler 480 SYBR Green I Master (Roche, Madison, WI, USA). Levels of mRNA were normalized relative to the levels of control ribosomal protein S28 (RPS28) mRNA (21). Data were analyzed by the Delta Delta Ct (2-ΔΔCT) method using Excel program. Primer sequences used were (forward/reverse): VEGF-α: 5′-AGT CCA ACA TCA CCA TGC AG-3′/5′-TTC CCT TTC CTC GAA CTG ATT T-3′, carbonic anhydrase 9 (CA9): 5′-TTT GCC AGA GTT GAC GAG G-3′/5′-AGC CTT CCT CAG CGA TTT C-3′, HURP: 5′-CAT TTT CCT TCA TAT TAT CAA TG-3′/5′-CAT TAT ATG CTA TAG AAG TGA ACA C-3′, and ribosomal protein S28 (RPS28): 5′-TTT TGG AGT CAG AGC GAG AAG-3′/5′-AGC ATC TCA GTT ACG TGT GG-3′.

### Preparation of Protein Extracts

Cells were washed with cold phosphate-buffered saline (PBS) and lysed in RIPA buffer. Cells lysates were sonicated on ice for 2 min with 30 s intervals followed by vortexing every 5 min during 45 min at 4°C. Lysates were centrifuged at 24,000 × *g* for 10 min. Supernatants were collected and saved at −80°C. For flash frozen tissue, prostate obtained from PCa patients (*n* = 8) and cystoprostatectomy patients (*n* = 4; used as control) was utilized. Cystoprostatectomy is a surgical procedure, which combines a cystectomy and a prostatectomy for the removal of bladder cancer tumors. Tissues were homogenized in an IKA Work tissue homogenizer (Wilmington, NC, USA). Proteins were extracted from the homogenate with the AllPrep DNA/RNA/Protein Mini Kit (Qiagen, Germantown, MD, USA) according to the manufacturer’s guidelines. Protein concentration was determined by Bradford assay (Bio-Rad, Hercules, CA, USA) using bovine γ-globulin (Pierce, Rockford, IL, USA) as standard.

### SDS-PAGE and Western Blot Analysis

Fifty micrograms of protein were separated in a 10.5–14% SDS-PAGE gradient gel, transferred to a nitrocellulose membrane, and incubated with blocking buffer containing primary antibodies (Santa Cruz Biotechnology, Santa Cruz, CA, USA) specific for HURP (diluted 1/400; sc-68540), β-actin (1/5000; Sc-8432), and glyceraldehyde-3-phosphate dehydrogenase (GAPDH, 1/5000; Sc-25778). The anti hypoxia-inducible factor 1-alpha [HIF-1α antibody (1/500)] was from R&D Systems (AF1935). Bound primary antibodies were visualized with HRP-conjugated antibodies specific for human IgG (diluted 1/1000 in blocking buffer; Abcam, Cambridge, MA, USA). After addition of a chemiluminescent substrate (Rockford, IL, USA), the membrane was immediately exposed on a CL-XPosure film (Thermo Fisher Scientific) and scanned with an Epson Perfection 4490 Photo scanner to detect bands. Relative intensities of the bands were quantified using ImageJ software (NIH online, Bethesda, MD, USA); the recorded values were normalized to the intensity of the respective β-actin signal.

### Immunohistochemistry

According to the manufacturer’s instruction provided in ABC Kit (Vector Laboratories Inc., Burlingame, CA, USA), immunohistochemistry (IHC) was performed on formalin fixed and paraffin embedded samples of benign prostatic tissues, low grade PCa, and high grade PCa. Briefly, 5-μm sections in thickness were deparaffinized and rehydrated followed by antigen retrieval with citrate buffer (pH 6.0) for 20 min. Endogenous peroxidase activity was quenched with 3% hydrogen peroxide for 10 min and unspecific bindings were blocked with 10% normal serum in room temperature for 1 h. Next, the slides were incubated with primary antibodies against HURP [rabbit polyclonal diluted 1/100 (cat # ab84509; Santa Cruz Biotechnology, Inc., Dallas, TX, USA)]; hypoxia-inducible factor 1-alpha (HIF-1α) [H-206, rabbit polyclonal diluted 1/50 (cat # sc-10790; Santa Cruz Biotechnology)]; heat-shock protein 60 (HSP60) [mouse monoclonal (LK1) diluted 1/50 (Abcam, Cambridge, MA, USA)]; and VEGF [VEGF (147), rabbit polyclonal diluted 1/50 (cat # sc-507; Santa Cruz Biotechnology)]. Sections were incubated overnight at 4°C. Negative controls were prepared by using a normal anti-rabbit IgG. After PBS wash, the slides were incubated with the components in ABC kits, and with 3,3 ′-diaminobenzidine (DAB) for color development. Finally, slides were counterstained in hematoxylin and mounted. Under microscope, subcellular localizations of IHC signals for each antibody were defined as membranous, cytoplasmic, or nuclear stain. The stain intensity was graded as no stain, weak, moderate, and strong.

### Methylation Assay

DNA (500 ng) from log growing LNCaP, DU-145, and PC3 cell lines was treated with sodium bisulfite and later purified using the EZ DNA Methylation Kit from Zymo Research (San Diego, CA, USA), as recommended. The bisulfite-treated DNA (BST-DNA) was denatured with 0.1 N NaOH for 10 min at room temperature and then amplified at 37°C for 24 h following Illumina’s recommended protocols, as we have previously published (22). The amplified DNA sample was then fragmented at 37°C for 1 h and precipitated with 2-propanol by centrifugation at 3000 × *g* for 20 min at 4°C. The DNA pellet was air dried, resuspended in buffer, and hybridized for 24 h at 48°C to the Illumina chips HumanMethylation27 to interrogate 27,000 CpG in more than 14,000 genes. A single-base extension protocol is followed by staining of the beadchip and several washing steps, as recommended by the manufacturer (Illumina). The chips were dried under vacuum for 55 min and scanned using the BeadArray reader (Illumina). The beta fraction (β value) of the HURP gene was obtained utilizing the GenomeStudio software v2011.1. For methylation analysis of HURP, we focused on cg 25465634-4010161.

### Bioinformatics Analysis for HURP Promoter

The promoter region of *DLGAP5/HURP* was studied for the binding sites of the transcription factor HIF using reported evidence (23, 24) on the minimal cis-regulatory elements required for HIF-dependent transactivation. Identified potential binding sites were analyzed in context of their location in methylated DNA regions. The possible binding sites were defined by those having at least 80% of nucleotide content identical to the reported canonical binding site for HIF (23, 24).

### Statistics

All reported values represent three independent cell culture experiments expressed as means ± SEM. Data for cell numbers, VEGF release, and mRNA expression were analyzed by two-way ANOVA (pO_2_ vs. day), followed by a *post hoc* Student–Newman–Keuls multiple comparisons test. Difference in HURP protein between tumor and normal tissue lysates were analyzed by the Mann–Whitney *U* test. A difference was considered significant at *p* < 0.05.

## Results

### Sensitivity to Hypoxia

**Figure 1:**
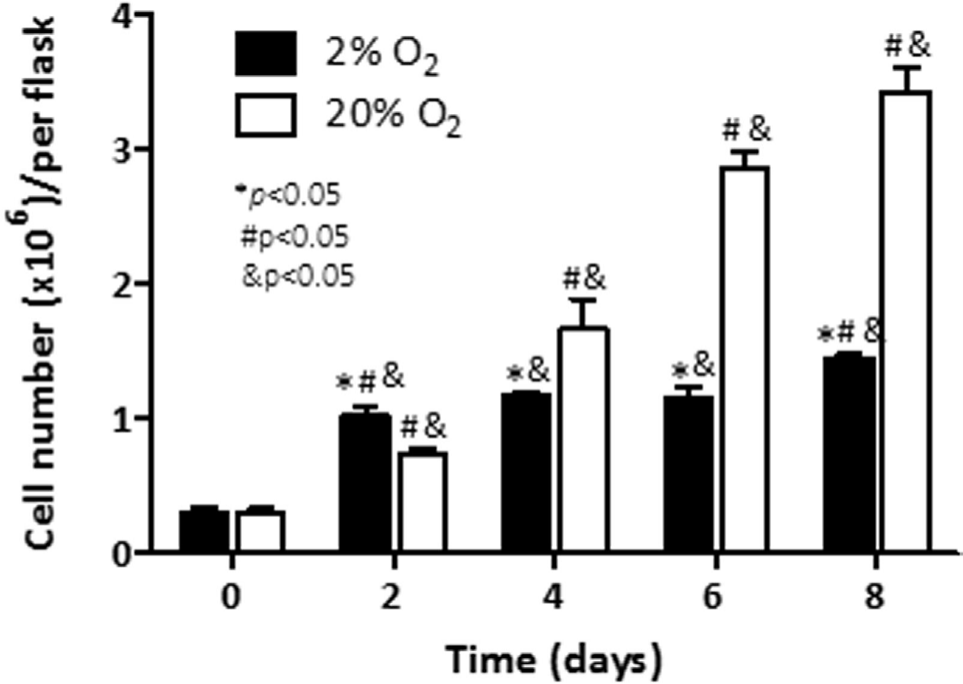
**Effect of hypoxia on cell viability: C4-2B cells were cultured at under hypoxia (■) or normoxia (□)**. Alive cells were counted in triplicate flasks using trypan blue exclusion to differentiate dead cells. *p < 0.05 when data at 2% O_2_ are compared to values at 20% O_2_ at the same time point; ^#^*p* < 0.05 when data at 2% O_2_ or 20% O_2_ are compared to a previous time point within the same O_2_%; and ^&^*p* < 0.05 when a specific data point is compared to the point at day 0 within the same O_2_%.

We first evaluated the effect of hypoxia on cell viability in C4-2B cells exposed to 20% O_2_ (normoxia) or 2% O_2_ (hypoxia). Accordingly, the number of living cells was counted in a time course experiment. Under normoxia (Figure 1), a significant increase of cell numbers was noted on day 2 (*p* < 0.05), and increased thereafter to reach a maximum on day 8, while under hypoxia, the increased cell numbers were markedly higher (threefold), when compared to the cell numbers on day 0 (*p* < 0.05). Numbers remained unchanged until day 6, and slightly increased by 20% at day 8. Cell numbers under normoxia, in contrast, did not experience further variation until completion of the experiment at day 8. Taken together, these findings show that C4-2B cells cultured under hypoxia have a significantly reduced growth kinetics relative to those cultured under normoxia.

**Figure 2:**
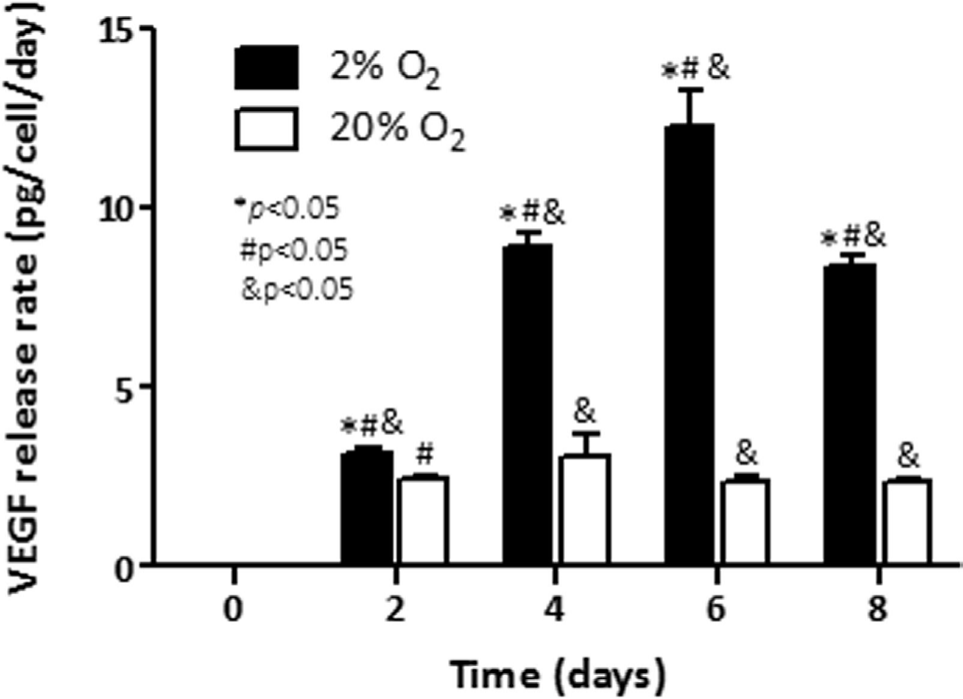
**Effect of hypoxia on VEGF production: rates of vascular endothelial growth factor (VEGF) release in conditioned culture media were measured by ELISA for C4-2B cells growing under hypoxia (■) or normoxia (□)**. *p < 0.05 when data at 2% O_2_ are compared to values at 20% O_2_ at the same time point; ^#^*p* < 0.05 when data at 2% O_2_ or 20% O_2_ are compared to a previous time point within the same O_2_%; and ^&^*p* < 0.05 when a specific data point is compared to the point at day 0 within the same O_2_%.

Rate of release of VEGF and the hypoxia-inducible pro-angiogenic factor associated with growth and aggressiveness (25) was next evaluated in cell culture supernatants by ELISA. As shown in Figure 2, under normoxia, the rate of released VEGF to the conditioned media was constant (2.5 pg/cell/day) up to day 8 (≈2.5 pg/cell/day). Cells cultured under hypoxia had constant induction until day 6. At that time point, a maximum release rate at day 6 (12.3 pg/cell/day) was noted. The release rate of VEGF decreased thereafter (*p* < 0.05) on day 8. At that last time point a release rate of 8.3 pg/cell/day was observed. The results indicate that exposure of C4-2B cells to low levels of oxygen enhances the production of the angiogenic factor, VEGF.

**Figure 3:**
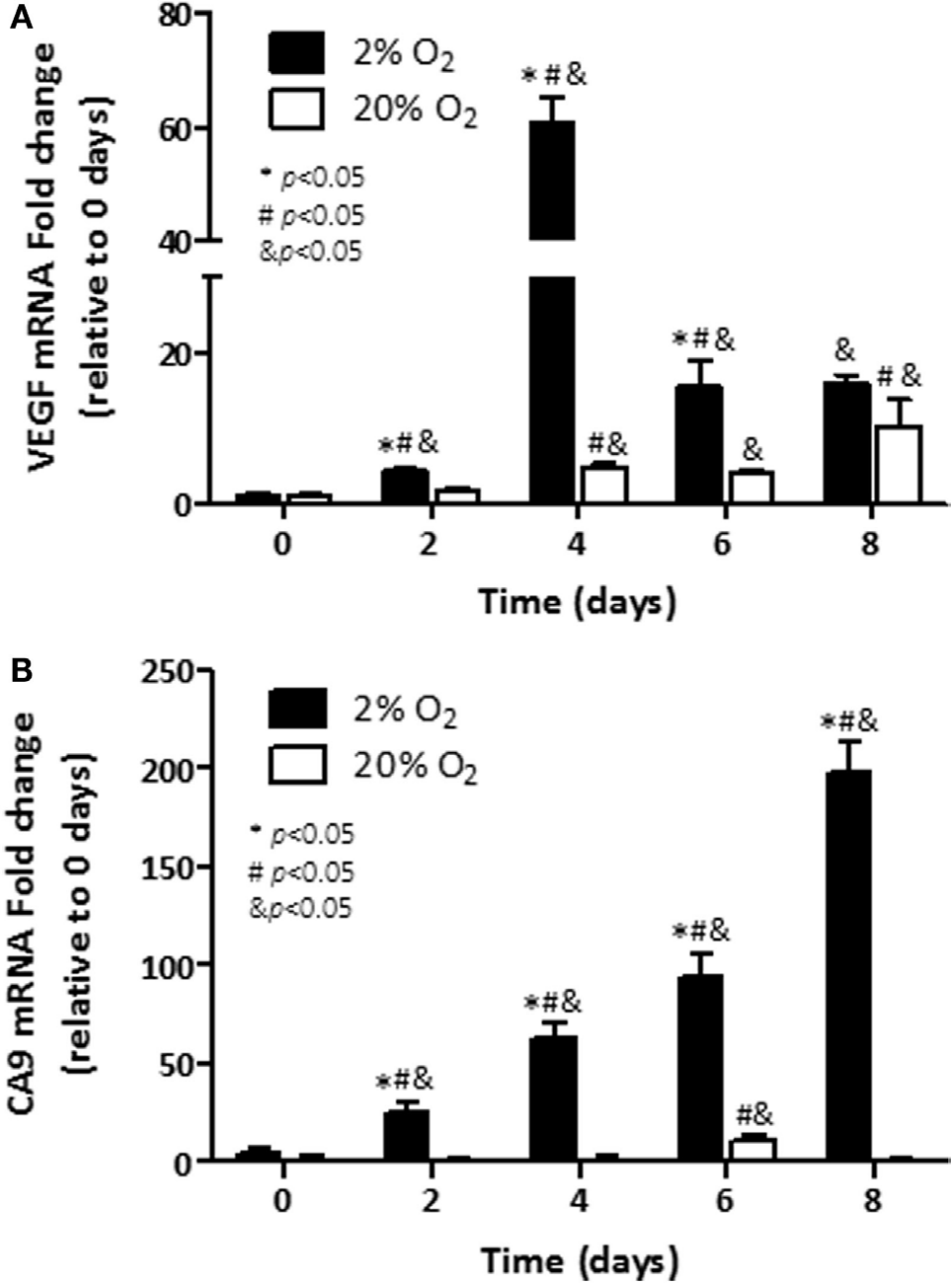
**Assessment transcripts of hypoxia-responsive genes: mRNA levels of; (A) Vascular endothelial growth factor (VEGF) and; (B) carbonic anhydrase 9 (CA9) were analyzed in C4-2B cells growing under hypoxia (■) or normoxia (□)**. *p < 0.05 when data at 2% O_2_ are compared to values at 20% O_2_ at the same time point; ^#^*p* < 0.05 when data at 2% O_2_ or 20% O_2_ are compared to a previous time point within the same O_2_%; and ^&^*p* < 0.05 when a specific data point is compared to the point at day 0 within the same O_2_%.

We next assessed whether transcriptional regulation of VEGF was associated with its production in cells cultured under hypoxia. Cells were incubated under normoxia or hypoxia over a period of 8 days, mRNA was extracted, and qRT-PCR was performed. The induction of VEGF expression (61-fold) was noted first on day 4 (*p* < 0.05) in cells growing under hypoxia, when compared with control cells (Figure 3A). In cells growing under normoxia, induction of VEGF transcripts was noted as well (*p* < 0.05). Particularly, on day 8, a 10-fold induction was evidenced. Observed levels in cells under normoxia at that time point were comparable to those noted in cells growing at 2% O_2_ (15.9-fold over base line).

Similar to VEGF, hypoxia-inducible metabolic enzyme CA9 is overexpressed in cancer cells (26) and has been proposed as a useful marker for hypoxic exposure (27). When measured, the transcripts of CA9 had a sustained induction over the 8 days of exposure to low oxygen (Figure 3B). On day 8, nearly 200-fold induction over baseline on day 0 was observed in hypoxic C4-2B cells (*p* < 0.05). In cells cultured under normoxia, no expression of CA9 was noted for most of the analyzed time course, but for 11.4-fold induction over basal levels, observed at day 6 (*p* < 0.05). In consistency with our recent publication (28) and reports from others (29, 30), our findings show that PCa cells exhibit features of increased aggressiveness when they are cultured under hypoxia.

### Effects of Oxygen Tension on the Expression of HURP

**Figure 4:**
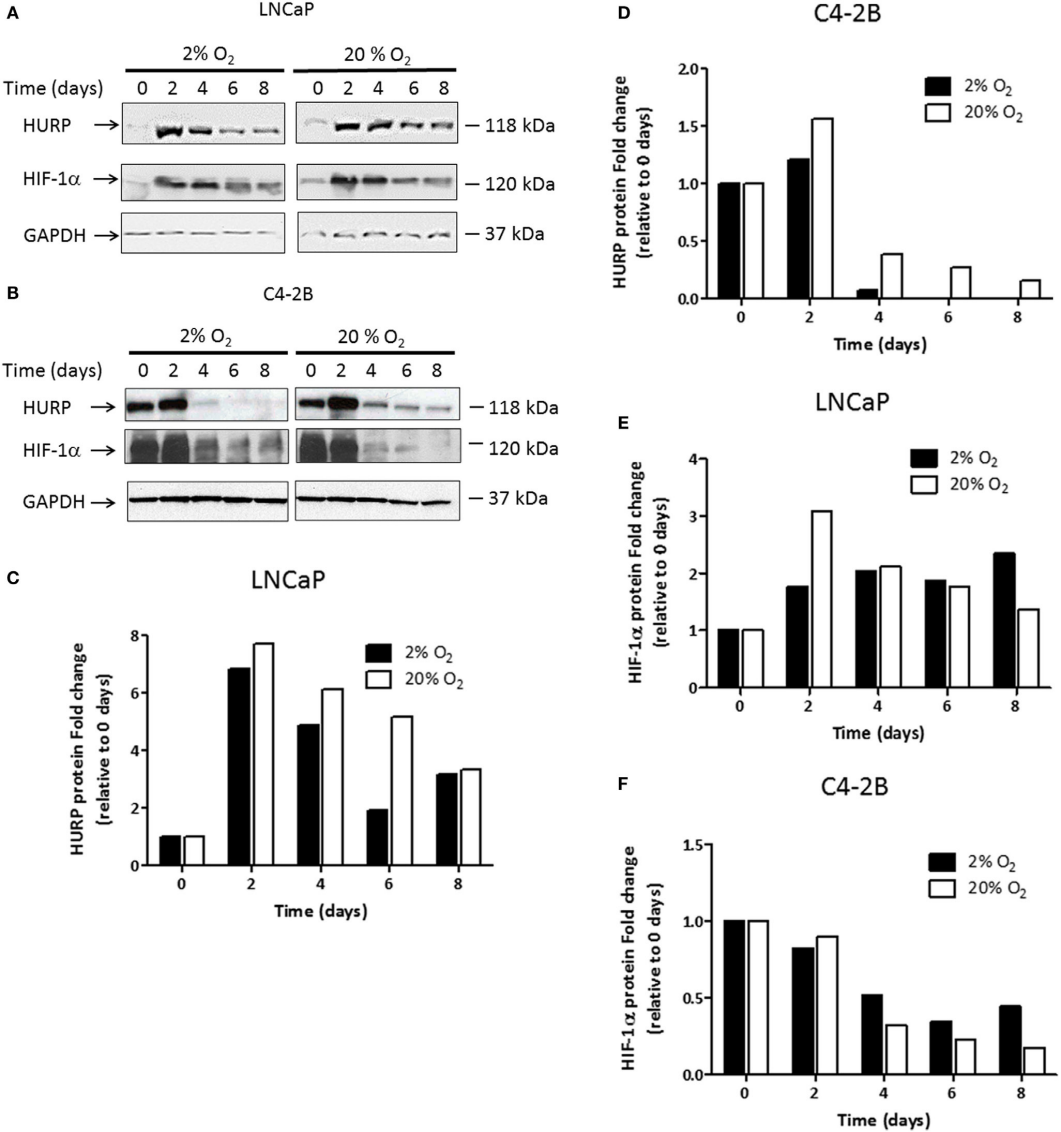
**Assessment transcripts of hypoxia-responsive genes: prostate cancer cells were cultured under hypoxia or normoxia during the indicated time intervals**. Protein lysates were prepared and 50 μg of the total protein was fractionated on the 12% SDS-PAGE, blotted onto the nitrocellulose membrane, and tested against antibodies. Western blot analysis using anti-hepatoma upregulated protein (HURP), hypoxia-inducible factor 1-alpha (HIF-1α), and glyceraldehyde-3-phosphate dehydrogenase (GAPDH) antibodies in **(A)** LNCaP cells and **(B)** C4-2B cells. The relative intensities of the bands under hypoxia (■) or normoxia (□) were quantified using the ImageJ software, and all the values were normalized to the intensities of the respective GAPDH signal. Data are expressed as the fold change obtained after dividing the optical density of HURP **(C,D)**; and HIF-1α **(E,F)** proteins for a given cell line and time point, relative to the OD observed of the respective protein under the corresponding value at 0 days.

We previously reported on the value of hypoxia-associated genes as prognostic markers of aggressive PCa (6). Among identified genes, transcripts of HURP independently predicted outcome in high-risk PCa (6). Because the role of hypoxia in the regulation of HURP expression is poorly understood, we set out to investigate the effect of varying oxygen levels on its expression. We assessed protein levels by Western blot analysis (Figures 4A,B) in C4-2B cells and their precursor cells, the LNCaP cell line (20). HURP protein levels were relatively low in LNCaP cells, compared to those detected in C4-2B at day 0. When quantified (Figures 4C,D), HURP protein increased to a similar extent at day 2 in LNCaP cells under normoxia (7.7-fold) and hypoxia (6.8-fold). In C4-2B cells, the increase was more pronounced in cells cultured under normoxia (1.6-fold) relative to those cultured at 2% O_2_ (1.2-fold). Irrespective of cell line and oxygen levels, HURP protein was drastically reduced over time. Under normoxia, the reduction of HURP expression was first noted at culture day 4 (21 and 61% decrease for LNCaP and C4-2B cells, respectively) and was reduced thereafter close to 90% over maximum induction, regardless of the cell line, on day 8. Under hypoxic conditions the reduction of HURP protein was more pronounced. This observation was particularly evident in the case of C4-2B cells; HURP protein was reduced by 93% on day 4 and was undetectable at days 6 and 8 in this cell line.

We next studied the expression level of HIF-1α protein (Figures 4A,B), a key regulator of cellular responses to variation in pO_2_. Expression level of HIF-1α quantified on day 2 (Figure 4E) was increased in LNCaP cultured under normoxia (3-fold) and hypoxia (1.8-fold). At that time point, HIF-1α protein was reduced (Figure 4F) by 10% in normoxia and 20% in hypoxia in C4-2B cells. From day 4, HIF-1α protein was considerably reduced under normoxic condition. In C4-2B cells, however, a more pronounced decrease (68% reduction at day 8), relative to that observed in LNCaP cells (55% reduction at day 8) was noted when the maximum level of HIF-1α for each corresponding condition was used as reference. The expression of HIF-1α was differentially affected by hypoxia in LNCaP and C4-2B cells. Following maximal expression at day 2 (1.8-fold), levels of expression of HIF-1α remained unchanged until day 8. C4-2B cells growing in hypoxia had 50% reduction in HIF-1α protein from base level after 4 days of culture; however, the decrease was not as pronounced as that observed in cells growing in normoxia. Under low oxygen level, HIF-1α protein levels remained unchanged (42% of base level) until culture day 8.

**Figure 5:**
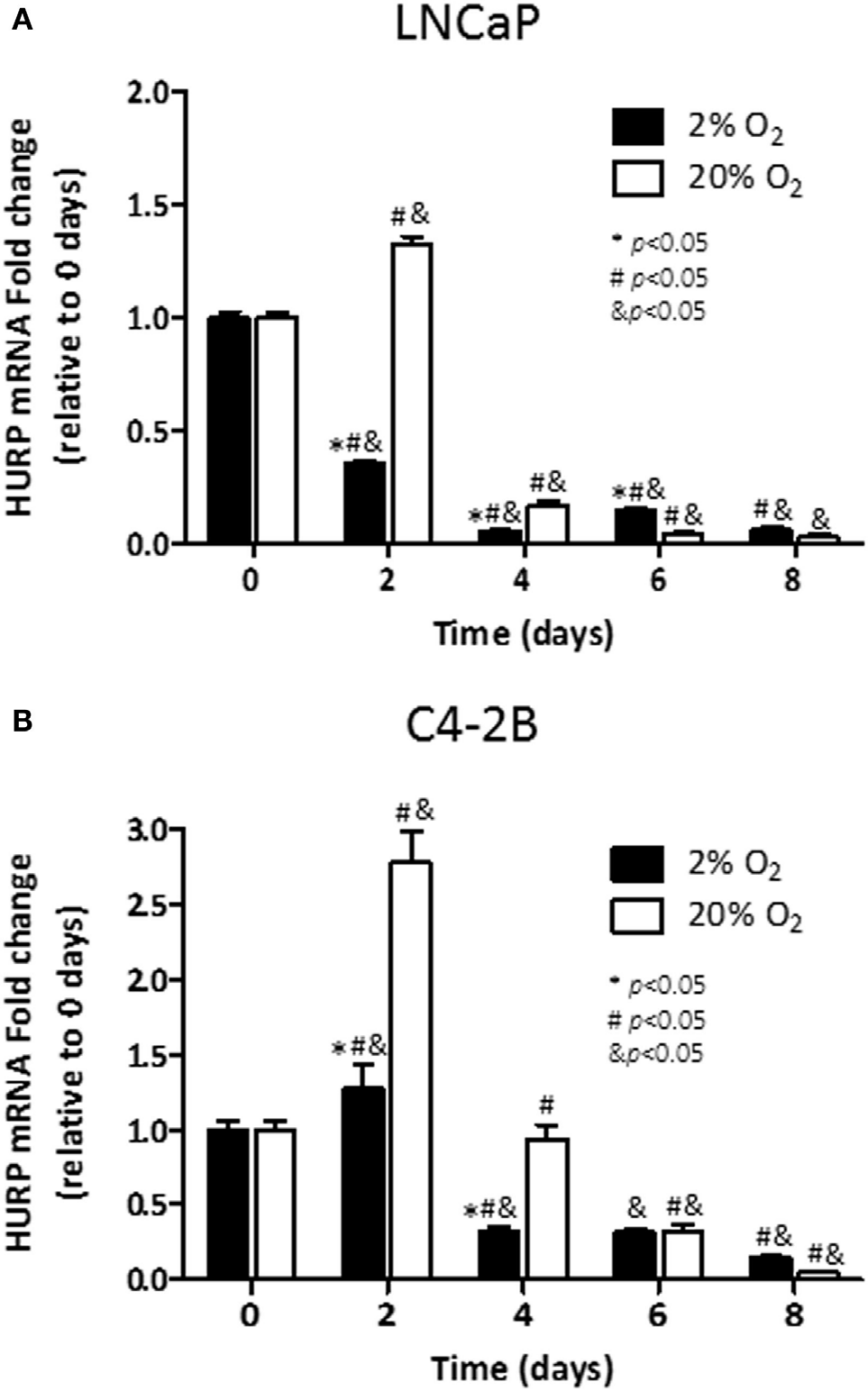
**Effect of hypoxia on HURP mRNA: mRNA levels of hepatoma upregulated protein (HURP) were analyzed in LNCaP (A) and C4-2B (B) cells growing under hypoxia (■) or normoxia (□) during 8 days**. *p < 0.05 when data at 2% O_2_ are compared to values at 20% O_2_ at the same time point; ^#^*p* < 0.05 when data at 2% O_2_ or 20% O_2_ are compared to a previous time point within the same O_2_%; and ^&^*p* < 0.05 when a specific data point is compared to the point at day 0 within the same O_2_%.

Next, we studied the transcriptional regulation of HURP under hypoxia using qRT-PCR analysis. In LNCaP cells, induction of HURP transcripts on day 2 (1.3-fold, *p* < 0.05) was evident for cells cultured under normoxic condition (Figure 5A). At that time point, cells growing under hypoxia had a 65% decrease in HURP transcripts (*p* < 0.05). From day 4 and thereafter, a pronounced reduction in HURP transcripts was noted irrespective of pO_2_ in LNCaP cells. Accordingly, at day 8, a decline of 98 and 94% over maximum levels was noted for cells growing under normoxia and hypoxia, respectively (*p* < 0.05). In accordance with protein expression data in C4-2B cells, early induction of mRNAs for HURP (Figure 5B) was noted on day 2. The increase in HURP transcripts observed at 20% O_2_ (3-fold, *p* < 0.05), was more pronounced than that noted in cells growing at 2% O_2_ (1.2-fold, n.s.). In C4-2B cells growing under normoxia, a reduction to baseline level was noted on day 4 (*p* < 0.05). At this oxygen level, additional decline in HURP transcripts was noted as function of time (*p* < 0.05), 68% on day 6 and 95% decrease on day 8. Under hypoxia, HURP transcripts increased by 27% on day 2 (*p* < 0.05), relative to the levels on day 0. Moreover, a time-dependent reduction of HURP expression was noted on day 4 (~70%), on day 6 (~70%), and on day 8 (85%). These findings suggest that HURP expression is associated to adaptive mechanisms of cellular response to varying oxygen tension.

### Levels of HURP Protein in PCa Tissues

**Figure 6:**
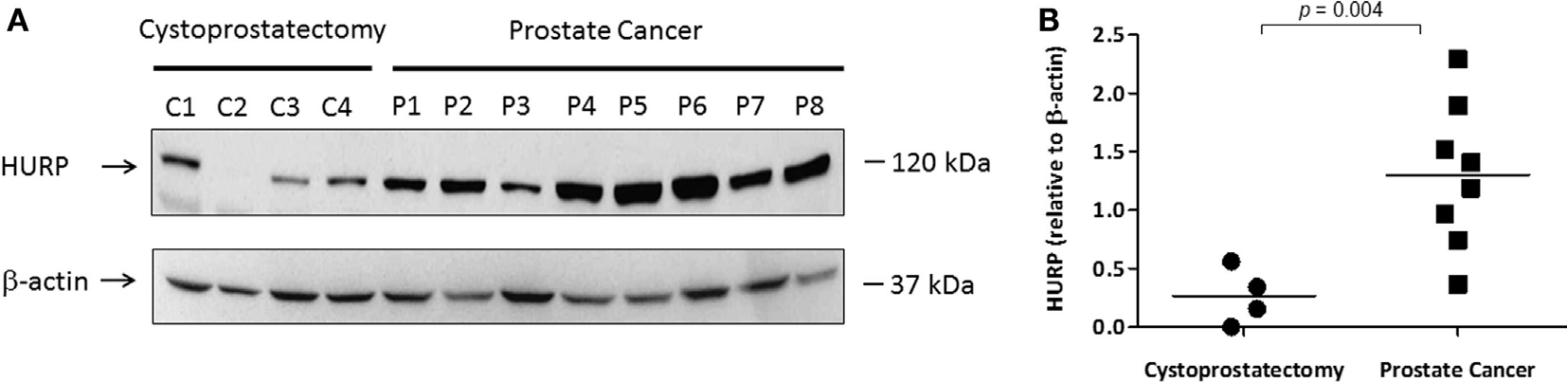
**Expression of HURP protein in PCa tissues**. Protein lysates (50 μg) extracted from frozen prostate tissues (C1–C4 samples from cytoprostatectomy patients and P1–P8 samples from PCa patients) were separated by SDS-PAGE. **(A)** Hepatoma upregulated protein (HURP) and beta (β)-actin levels in the samples were identified by Western blot as described in Section “Materials and Methods.” **(B)** The results were normalized to β-actin. Normalized ratios were compared between controls and PCa tumors. The level of significance was set at *p* < 0.05 between PCa and cytoprostatectomy samples.

Based on our publication regarding the prognostic value of HURP in aggressive PCa (6), we next explored the expression of HURP protein in PCa tumors. For this purpose, we prepared protein lysates from PCa tumors obtained from patients selected over the basis of their HURP mRNA expression and analyzed them by Western blot. Prostatic tissue was obtained from patients suffering with PCa or cystoprostatectomy (controls). Separated proteins were transferred onto a nitrocellulose membrane and blotted against anti-human HURP antibodies. The level of HURP protein (Figure 6A) was analyzed by Western blot. Densitometry analysis revealed almost fivefold higher relative in PCa tissue relative to control (cystoprostatectomy) tissue (*p* < 0.05) (Figure 6B). These data suggest that protein expression for HURP can be elevated in PCa tumors at the protein level.

### Histological Localization of HURP in the Context of Hypoxia-Responsive Molecules

**Figure 7:**
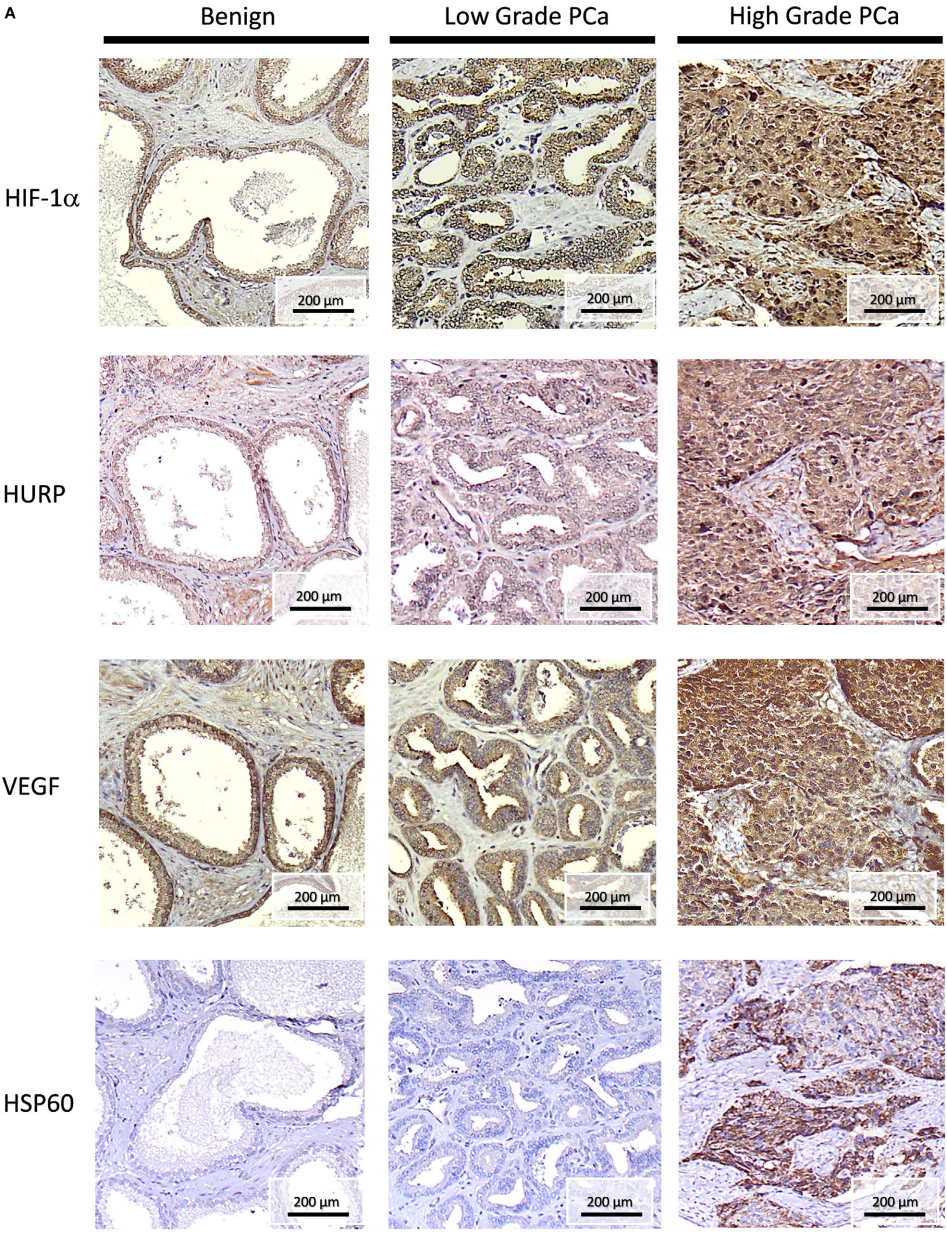

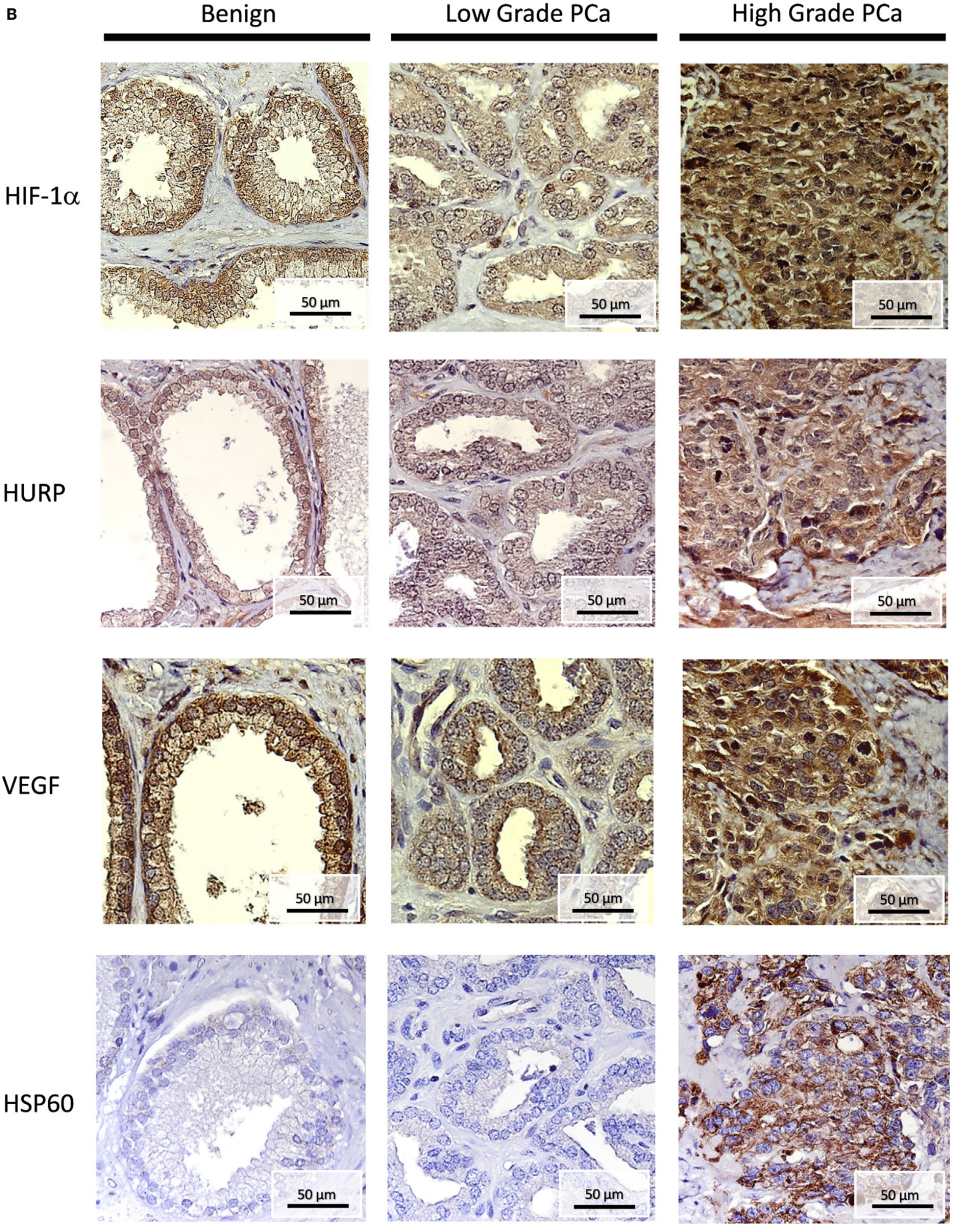
**Histopathology of HURP and hypoxia-sensitive molecules in association with prostate cancer progression**. Hepatoma upregulated protein (HURP), hypoxia-inducible factor 1-alpha (HIF-1α), Vascular endothelial growth factor (VEGF), and Heat-shock protein 60 (HSP60) immunostaining was assessed in benign tissues, Gleason <7 low grade PCa, and Gleason >7 high grade PCa tumors. Slides were counterstained in hematoxylin and mounted. Representative images of HURP, HIF-1α, VEGF, and HSP60 expression in bening tissue, low grade and high grade PCa tissues are shown. **(A)** Analysis of immunostaining intensity. Under a 100× magnification, the stain intensity was graded as no stain, weak, moderate, and strong stain. **(B)** Subcellular localization is shown under a 400× magnification.

We next performed IHC in FFPE tissue blocks obtained from benign, low grade, and high grade PCa, as shown in Figure 7A (low magnification, 10×) and 7B (high magnification, 40×). IHC signals for HURP were moderate in nuclei staining and weak in cytoplasm localization of both benign prostatic epithelia and low grade PCa. High staining was found in the nuclei and cytoplasm of high grade PCa. Since expression of hypoxia-regulated molecules has been found associated with pathology and aggressive phenotype in PCa (3, 29, 31), we next evaluated their histological localization in relation with tumor grade. Similarly to HURP, IHC signals for HIF-1α were moderate in nuclei, and weak in cytoplasm of benign prostatic epithelia, moderate in both cytoplasm and nuclei of low grade PCa, and strong in both nuclei and plasma of high grade PCa. The immune intensity of VEGF, a HIF-1α induced protein, was also analyzed. IHC signals for VEGF were strong in nuclei and moderate in cytoplasm of benign prostatic epithelia, moderated in both nuclei and cytoplasm of low grade PCa, and strong in both nuclei and cytoplasm of high grade PCa. Finally, histological expression of HSP60, the mitochondrial chaperonin, actively involved in the accumulation of HIF-1α (32) was also analyzed. The pattern of IHC signals for HSP60 was different to that of HIF-1α, HURP, and VEGF: IHC signals were not present in benign prostatic epithelia and in low grade of PCa, and not present in nuclei of high grade PCa cells. However, staining was strong in cellular membrane and cytoplasm of high grade PCa.

### Methylation of HURP Promoter Is Associated with the Decrease of HURP Expression in PCa Cell Lines

**Figure 8:**
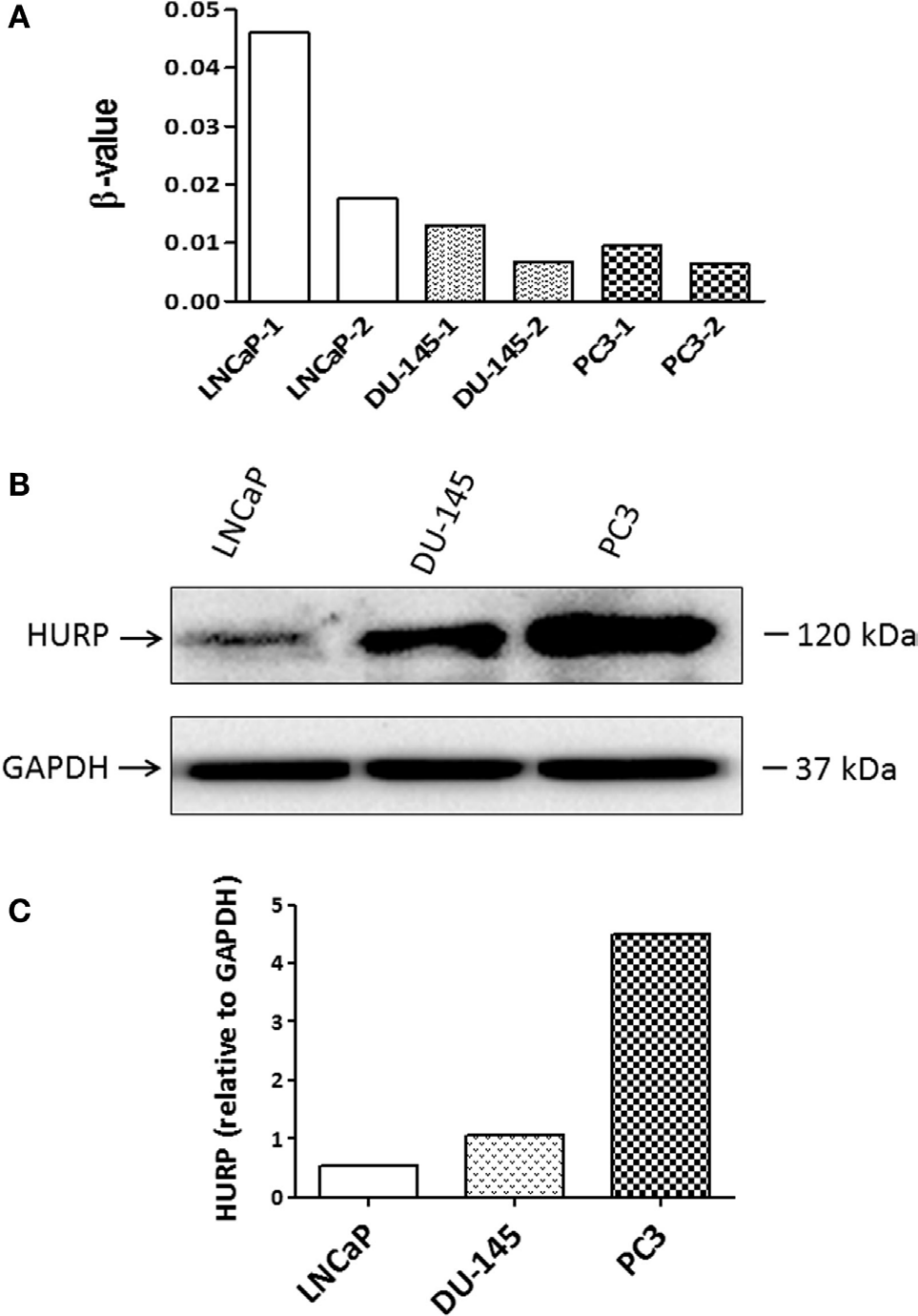
**Methylation of HURP promoter and HURP expression in PCa cell lines**. **(A)** Epigenetic analysis of the promoter of HURP demonstrated the methylation status in LNCaP, DU-145, and PC3 cells in two independent experiments per cell line. **(B)** Protein lysates (50 μg) extracted from whole cell lysates were separated by SDS-PAGE. Hepatoma upregulated protein (HURP) and glyceraldehyde-3-phosphate dehydrogenase (GAPDH) levels in the samples were identified by Western blot as described in Section “Materials and Methods.” GAPDH was used as internal control for loading and transfer. **(C)** The relative intensities of the bands were quantified using the ImageJ software, and all the values were normalized to the intensities of the respective GAPDH signal.

Aberrant DNA methylation is a common epigenetic aberration in PCa and has led to the identification of markers for disease diagnosis and prognosis (33). To determine whether an epigenetic mechanism contributes to the downregulation of HURP expression in PCa cells, DNA methylation status at the promoter region of the HURP gene was examined in the PCa cell lines LNCaP, DU-145, and PC3. Analysis of the methylation status of the promoter for two independent experiments in the cell lines revealed a significant increase of the methylation level of the promoter of HURP (cg 25465634-4010161) in LNCaP cells, when compared to the methylation level noted in DU-145 or PC3 cell as shown in Figure 8A. Interestingly, the noted increase of the promoter methylation was associated with decrease of protein level of HURP (4.5, 1.1, and 0.5 relative density units for PC3, DU-145, and LNCaP, respectively) as evidenced by densitometry analysis of the Western blot signals (Figures 8B,C).

### Analysis of HURP Promoter for HIF-1-Binding Sites

To show whether the regulation of *DLGAP5/HURP* gene expression is HIF-1-dependent, we analyzed the putative HIF binding sites on the promoter of *DLGAP5/HURP* gene. The analysis of the non-translated region upstream from the start codon (9057 nt), using the 5′-rcgtg-3′ (*R* = g/a) motif identified by Wenger et al. (24), revealed four putative HIF binding sites, 5′-GCGTG-3′ at position: −8310; 5′-ACGTG-3′ at position: −3815; 5′-ACGTG-3′ at position: −2616; and 5′-GCGTG-3′ at position: −1134 upstream from the start codon (+1). The positions of the identified binding sites are outlined in Table 1. This information suggests an essential role for HIF-1 transcription factor in the transcriptional activation *DLGAP5/HURP* promoter in response to hypoxia.

**Table 1:** **Identified putative HIF-1-binding sites in the promoter sequence of the *DLGAP5* gene**.

Upstream position	Sequence (5′–3′)
8310–8306	GCGTG
3815–3811	ACGTG
2616–2612	ACGTG
1134–1130	GCGTG

## Discussion

We investigated the effect of hypoxia on the expression of HURP in human PCa cells. In the C4-2B cell line hypoxia, as expected, modified cell viability and increased the rate of release and mRNA expression of VEGF, the pro-angiogenic factor. Likewise, mRNA expression of CA9, a marker for hypoxic exposure and tumor aggressiveness, was highly induced in response to low pO_2_. Both Western blot and mRNA expression analyses in LNCaP and C4-2B cells showed that HURP expression was reduced by time of culture in normoxia. Hypoxia, however, accelerated the rate of decrease of HURP expression. In contrast to what was observed *in vitro*, HURP expression was increased in PCa tumors, and its elevated expression seems to be associated with tumor grade. Overall, our studies suggest that tumor-associated hypoxia is a relevant determinant of the expression of HURP in PCa cells.

We and others have demonstrated the effects of low pO_2_ on viability responses in cultured PCa cells (28, 34–40). In agreement with our previous findings (28) the C4-2B subline, a derivative of the LNCaP cell line, grew slower in hypoxia than cells cultured under normoxia (Figure 1). The methodology for assessment of cell viability was validated by us in the initial phase of our study. We used different cell densities and various timelines including the one referred herein (8 days) or even longer time points. As expected, effects on cell numbers and VEGF production were proportional to cell densities. Although our studies have the limitation of the lack of information regarding the effect of oxygen on cells of those with prostatic carcinoma origin, they add to the information indicating the effect of low pO_2_ on the viability responses in cultured PCa cells.

The adaptive response of PCa cells to a changing metabolism and survival environment has been considered a relevant factor of tumor aggressiveness. A component of the response of cancer cells to hypoxia is VEGF. Since hypoxia promotes secretion of this pro-angiogenic factor (30, 41, 42), assessment of its release to the culture medium can be used as an indicator of low pO_2_. C4-2B cells cultured under low pO_2_ showed increased VEGF release rate. The increase in VEGF secretion rate was transcriptionally driven, as it could be expected (30, 41, 42). The hypoxia-inducible enzyme CA9, overexpressed in cancer cells (26), has been proposed as a marker for hypoxic exposure and tumor aggressiveness (27). Accordingly, we demonstrated that under hypoxic culture CA9 transcripts were elevated nearly by 200-fold in hypoxic C4-2B cells, when compared to cells growing under normoxic conditions. All together these results show that, in agreement to reported findings obtained in other cell lines (28, 43), the C4-2B subline shows characteristics of increased aggressiveness when cultured under hypoxia.

In a previous report, using a validated data mining approach (21, 44–46), we identified hypoxia-associated genes that can be utilized as markers of aggressive PCa (6). Among these genes, transcripts of HURP were associated with Gleason score and systemic progression. We further validated HURP as an independent outcome predictor in high-risk PCa (6). Our published data supported the association of the hypoxic transcriptome and PCa, and provided evidence to sustain the participation of hypoxia-associated genes into the mechanisms of PCa progression. To further support this hypothesis, we scrutinized the promoter region of HURP for binding sites of HIF-1α (Table 1). We found four putative HIF binding sites upstream of the HURP transcription start.

Most of the identified binding sites for HIF-1α on the promoter of HURP reside in highly methylated regions, known to be commonly inversely correlated with gene expression and gene methylation in cancer cells (47), including PCa cell lines (48). In support of a notion for the regulatory effects of promoter methylation on HURP expression, we found that the increased methylation of HURP promoter (Figure 8A) is associated with a reduction in protein levels in the cell lines LNCaP, DU-145, and PC3 (Figure 8B). Epigenetic modification of the HURP promoter, therefore, correlates with reduction of HURP expression. Although the data does not include hypoxia as an experimental variable, these findings suggest that the methylation of HURP promoter is responsible for the reduction of the basal expression of HURP in PCa cell lines. It may be anticipated that low oxygen, a condition known to increase tumor aggressiveness will have regulatory effects on HURP expression. Increased methylation of promoters can repress gene expression by directly preventing binding of transcription factors (49). It may be plausible to suggest that methylation of HURP promoter may prevent the binding of HIF to its promoter region. As a consequence, inhibition of HURP expression may occur. This highly speculative hypothesis is interesting since it suggests a regulatory effect of methylation in silencing HURP gene expression in PCa cells exposed to hypoxia. Future studies are necessary to directly prove the importance of HIF-1α binding sites on HURP promoter, or that of other signaling factors associated with cellular responses to hypoxia, on the regulation of HURP expression under hypoxia condition. Utilization of epigenetic drug treatment of PCa cell lines followed by gene expression analysis for HURP may allow us to assess the effects of pO_2_ on the expression of HURP. Those experiments deserve special attention due to the reported effects that promoter methylation exerts on expression of hypoxia-controlled genes (e.g., CA9, studied herein) in context of tumor microenvironment (23).

Analysis of protein levels of HIF-1α showed that expression of this transcription factor, essentially involved in the cellular response to hypoxia, was observed in PCa cells cultured under normoxia. Those findings are consistent with literature showing that HIF-1α is expressed in normoxic conditions as a means to carry out a regulatory role in response to regulatory factors such as cytokines, hormones, and genetic alterations (50, 51). Independent of the cell line, HIF-1α expression was maintained at a higher level under prolonged (8 days) hypoxia. Elevated and sustained HIF-1α expression *via* a non-transcriptional mechanism has been demonstrated to block DNA replication (52). By binding to components of the pre-replicative complexes that assemble at origins of replication, HIF-1α inhibited the activation of minichromosome maintenance helicase, consequently hindering unwinding of the DNA during replication (52). It is likely, that the transcription-independent mechanism of cell cycle arrest in response to hypoxia may be operating in the studied PCa cell lines. Additional experiments are needed to clarify this point.

We analyzed protein expression of HURP in tumors and made associations with the expression of hypoxia-associated molecules. As a follow-up of our report showing increased transcripts of HURP in association with increase in the Gleason score and systemic progression (6), the level of HURP protein analyzed by Western blot (Figure 6) and IHC (Figures 7A,B) was higher in tumors relative to control tissue. These results agree with a previous study showing elevated expression of HURP protein in fine needle cell aspirates obtained from hepatocellular carcinoma patients (53). In the cited study, further analysis revealed association between positive HURP staining and a shorter disease-free survival (53). In our case, analysis of larger number of samples will allow us to establish an association between HURP expression and aggressive PCa. This study is certainly needed given the inter-individual heterogeneity in HURP expression noted by us in non-tumor tissue as well as in prostate carcinomas. We cannot provide explanation for those findings at this point. However, we suggest that histological observation is certainly a good complement to Western blot analysis. Histological techniques helped us to reveal with higher sensitivity distinct features for tissue expression of HURP not clearly revealed by Western blot analysis.

Our results in PCa tumors, however, somehow, contradict evidence in PCa cell line cultures showing that HURP expression was reduced as a function of incubation time and hypoxia. We attribute the apparent discordant results in part to the presence of complex interactions of the tumor microenvironment observed in tumors (54). Equally relevant is the variability provided by the well-known heterogeneity in PCa (55). A reduction in HURP expression in relation with incubation time is expected based on the cell cycle-associated nature of this protein (13–15). We still do not have direct evidence experimental of the direct role of hypoxia in HURP’s expression and the status of other signaling pathways involved in its regulation. Despite of the lack of information, the literature has illustrated pathway-specific differences between tumor cell lines and tumor cells (56). Use of pathway-specific enrichment analysis of publicly accessible microarray data and quantified the gene expression differences between cell lines, tumor, and normal tissue cells for different tissue types including PCa, revealed substantive numbers of genes and associated pathways common between cell lines and tumor cells (56). In that study, however, a fraction of pathways showed expression profiles that differed significantly between cell lines and tumors included cell cycle and a number of metabolic and transcription-related pathways (56). Among them, metabolic pathways closely sensitive to variation of the tumor microenvironment (e.g., ATP synthesis, oxidative phosphorylation, pyrimidine and purine metabolism, and proteasome) were significantly altered in cell lines compared to tumors (56). Over the basis of those results, we can postulate that characteristics of the tumor microenvironment, including presence of metabolites, cell-to-cell interaction in the multilayered structure of solid tumors, among many others may be particularly relevant for the expression of HURP in tumors. Differences in environmental selection pressure between *in vitro* culture and tumor tissue may help to explain, in part, our dissimilar results.

Hypoxia in tumor regions exists due to multiple factors, such as low blood irrigation, aberrant angiogenesis, and excessive oxygen consumption by cancer cells. The hypoxic areas are characterized by variable blood flow and pO_2_. In common with other solid tumors, pO_2_ in PCa fluctuates, resulting in acute and chronic hypoxia (3). Mostly because of the conflicting information showing lack of correlation between pO_2_ values measured in the PCa nidus and clinical outcome (3), assessing pO_2_ in PCa tissue is at preliminary stages. A number of publications have indicated a correlation of hypoxia-associated molecules, such as VEGF, HIF-1α, osteopontin, lysil oxidase, and glucose transporter-1 with pathology and patient features in PCa [reviewed in Stewart et al. (3)]. Therefore, hypoxia-associated molecules are reliable as subrogates of hypoxia and indicators of aggressive tumors. In agreement with this notion, tumor expression of HURP, along with expression of HIF-1α, VEGF (a HIF-1α target), and HSP60 (involved in the regulation of HIF-1α protein stability) were associated with PCa progression. It is therefore likely that the relationship between expression of HURP and hypoxia-responsive molecules that we observed *in vitro* has a histological counterpart in PCa tumors. We postulate that localization of HURP protein in PCa tumors should be further explored as subrogate of tumor hypoxia and as marker of aggressive disease.

## Conclusion

The role of pO_2_ in PCa pathobiology has been underappreciated. Tumor-associated hypoxia has been associated with malignant progression, metastasis, resistance to therapy, and poor clinical outcome. Among hypoxia-associated proteins, HURP has multifunctional biological properties, which are compatible with its role in carcinogenesis. A direct cause and effect relationship between hypoxia and HURP expression is yet to be established. Nevertheless, our findings bring insight into the effect of low oxygen on expression of HURP in PCa. Additionally our results provide basis for utilization of tumor-associated molecules as relevant tumor markers. The expression of HURP, together with that of hypoxia-responsive molecules, such as HIF-1α, VEGF, and HSP60, may serve as predictor of aggressive tumors.

## Author Contributions

IE performed experiments, drafted the manuscript, edited, and approved the final version; MS performed experiments, drafted the manuscript, edited, and approved the final version; TM performed experiments and approved the final version; LF assisted with pathological data analysis and approved the final version; MH contributed to conception and design, edited and approved the final version; XZ performed experiments, wrote the manuscript, edited, and approved final version; JZ performed experiments, edited, and approved the final version; CG contributed to conception and design, wrote the manuscript, edited, and approved final version.

## Conflict of Interest Statement

The authors declare that the research was conducted in the absence of any commercial or financial relationships that could be construed as a potential conflict of interest.

## Abbreviations

CA9, carbonic anhydrase 9; CO_2_, carbon dioxide; DLGAP5, discs, large (*Drosophila*) homolog-associated protein 5; DNA, deoxyribonucleic acid; ELISA, enzyme-linked immunosorbent assay; HIF-1α, hypoxia-inducible factor 1-alpha; HIFs, hypoxia-inducible factors; HSP, heat-shock protein; HURP, hepatoma upregulated protein; IHC, immunohistochemistry; mRNA, messenger ribonucleic acid; O_2_, dioxygen; PCa, prostate cancer; pO_2_, oxygen tension; qRT-PCR, reverse transcription polymerase chain reaction; RNA, ribonucleic acid; VEGF, vascular endothelial growth factor.
